# Smoking trends in Poland (2012–2021)—insights from the comprehensive cardiovascular risk prevention program

**DOI:** 10.3389/fpubh.2025.1643059

**Published:** 2025-09-03

**Authors:** Grzegorz Kubielas, Izabella Uchmanowicz, Christopher S. Lee, Michał Czapla, Grzegorz K. Jakubiak, Stanisław Surma, Magdalena Lisiak, Marta Wleklik

**Affiliations:** ^1^Department of Nursing, Faculty of Nursing and Midwifery, Wroclaw Medical University, Wrocław, Poland; ^2^Department of Health Care Services, Polish National Health Fund, Central Office in Warsaw, Warsaw, Poland; ^3^Centre for Cardiovascular Health, Edinburgh Napier University, Edinburgh, United Kingdom; ^4^Boston College William F. Connell School of Nursing, Chestnut Hill, MA, United States; ^5^Department of Emergency Medical Services, Faculty of Nursing and Midwifery, Wroclaw Medical University, Wrocław, Poland; ^6^Group of Research in Care (GRUPAC), Faculty of Health Science, University of La Rioja, Logroño, Spain; ^7^Institute of Heart Diseases, University Hospital, Wrocław, Poland; ^8^Department of Pharmacology, Faculty of Medical Sciences in Zabrze, Medical University of Silesia, Zabrze, Poland; ^9^Department of Internal Medicine and Clinical Pharmacology, Medical University of Silesia, Katowice, Poland

**Keywords:** smoking cessation, tobacco use disorder, cardiovascular disease prevention, public health intervention, sex differences

## Abstract

**Background:**

Understanding smoking prevalence trends across demographic groups is crucial for effective public health interventions. Monitoring such trends helps tailor prevention strategies and allocate resources to the most affected populations. These insights support the design of more equitable and effective tobacco control policies.

**Aim:**

This study analyzes data from the Comprehensive Cardiovascular Risk Prevention Program (CCRPP) in Poland (2012–2021) to assess smoking trends by age and gender among program participants.

**Methods:**

A repeated cross-sectional observational design was used to analyze data collected from 2012 to 2021, including participants who met the predefined age criteria (35, 40, 45, 50, or 55 years) at the time of enrollment, based on their birth year. Smoking prevalence was examined across age groups and sex to identify trends and disparities.

**Results:**

Smoking prevalence significantly declined from 28.91% in 2012 to 21.77% in 2021 (*p* < 0.001). The largest absolute reduction was seen in the 45-year age group (from 29.90 to 19.50%, *p* < 0.001). Across all age groups, men consistently had higher smoking rates than women, with the gap most pronounced in the 55-year group in 2012 (41.01% vs. 30.08%, *p* < 0.001). Although smoking rates decreased over time in both sexes, this male predominance persisted and remained statistically significant (*p* < 0.001).

**Conclusion:**

Smoking prevalence declined significantly among participants of the CCRPP, particularly in middle-aged individuals. Persistent sex differences highlight the need for more targeted smoking cessation interventions tailored to men. The CCRPP’s large-scale, standardized data collection offers a valuable platform for monitoring national smoking trends and informing future tobacco control policies in Poland.

## 1 Introduction

Tobacco smoking remains one of the most important modifiable health risk factors (1); therefore, taking action to promote smoking cessation is one of the most important public health challenges. It is worth emphasizing that passive exposure to tobacco smoke is an equally important problem (2).

Tobacco smoke contains numerous carcinogens that affect various signal transduction pathways (3). Exposure to tobacco smoke is associated with increased heart rate, systolic blood pressure, myocardial contractility, and cardiac output (4), as well as development of atherosclerosis through an increase in oxidative stress (5), endothelial dysfunction (6), inflammation (7), thrombosis (8), and platelet activation (9).

Cardiovascular diseases remain the leading cause of morbidity and mortality worldwide (10, 11). Notably, only smoking cessation, not reduction, is associated with a significant reduction in cardiovascular risk (12). In 2017, the multi-center National Population Health Examination Survey (WOBASZ) results were presented. This study, conducted between 2003 and 2014, found that although the percentage of people smoking tobacco was decreasing, it still remained significant and above targeted prevalence (13). More recent data collected retrospectively on a large population in 2016–2020 were presented in 2022 (11.6% of women and 17.1% of men declared smoking). However, this study involved only professionally active adults (14).

There is insufficient data available on tobacco smoking in Polish society, therefore conducting scientific research that can contribute to obtaining additional data is very important. Expanding knowledge about the current epidemiology of tobacco smoking remains essential, taking into account the influence of gender and age for optimal identification of the group of people who should be covered by intensified educational and preventive activities. It should be emphasized that age and gender are factors that significantly influence cardiovascular risk [10.1016/j.atherosclerosis.2023.117269; 10.1016/j.cjca.2021.02.005; 10.3390/jcm14051444], hence, additionally, understanding the relationship with tobacco smoking is crucial in undertaking initiatives aimed at preventing cardiovascular diseases (15–17).

One of the initiatives addressing cardiovascular risk factors in Poland was the Comprehensive Cardiovascular Risk Prevention Program (CCRPP, org. ChUK), a nationwide cardiovascular prevention project conducted between 2012 and 2021 within primary healthcare services. The program aimed at early detection and management of cardiovascular risk factors, including smoking, through comprehensive screenings and preventive interventions. The program is addressed to middle-aged adults (35–65 years old), without diagnosed cardiovascular disease, diabetes, chronic disease, or familial hypercholesterolemia. As part of the program, blood pressure, blood glucose, and lipid profile determination as well as basic anthropometric measurements were performed. The CCRPP provides a unique opportunity to examine long-term smoking trends and their association with cardiovascular risk across different demographic groups from the entire Polish society (18). During similar period, other, smaller-scale cardiovascular disease prevention programs were also conducted. One example is KORDIAN: the nationwide prevention program for atherosclerosis and heart disease through education of people with elevated cardiovascular risk factors (19). Similar projects are also being conducted in other countries around the world, such as the National Public Health Prevention Programme in Greece (20) and Million Hearts 2022 or Well-Integrated Screening and Evaluation for Women Across the Nation (WISEWOMAN) in the United States (21, 22).

The purpose of this study was to analyze a decade of data (2012–2021) from the CCRPP to assess smoking trends by age and gender among program participants.

## 2 Methods

### 2.1 Study population

This study utilized a repeated cross-sectional observational design to analyze cross-sectional data collected annually from 2012 to 2021. The total sample size across all years was 880,136 participants, including 342,802 men (39.0%) and 537,334 women (61.0%). In 2012, the initial sample size was 83,974 participants, which declined to 44,797 by 2021. Participants, drawn from the Polish population, were enrolled in the CCRPP based on predefined age criteria (35, 40, 45, 50, or 55 years), determined by birth year, and were stratified by sex. Eligibility criteria included: age between 35 and 65 years; no prior diagnosis of diabetes mellitus, chronic kidney disease, familial hypercholesterolemia, or cardiovascular disease; and no participation in the CCRPP within the past 5 years (23). Data collection methods were standardized annually to ensure methodological consistency. Participants were invited through primary care physicians during routine visits or were recruited via local health initiatives. Smoking status was assessed according to the CCRPP criteria, defining a smoker as an individual who reported current use of tobacco products or regular smoking within the previous 12 months. Smoking status was recorded during a structured interview conducted by trained healthcare staff, with additional information collected on the number of cigarettes smoked per day and years of smoking. Data were entered into a uniform national electronic template (SIMP) and transferred to the National Health Fund database, ensuring methodological consistency across all participating sites and study years (23).

### 2.2 Ethical

All procedures were conducted in compliance with the relevant guidelines and regulations. Ethical review and approval were not required for this study, as no identifiable data were collected, nor were any laboratory tests or medical interviews conducted. Consequently, approval from a bioethics committee was not necessary under Polish national legislation.

### 2.3 Statistical analysis

The comparison of categorical variables between groups was performed using the chi-square test (with Yates’ correction for 2×2 tables) or Fisher’s exact test when low expected frequencies were observed in contingency tables. Smoking prevalence (%) was calculated annually for each sex and age group and used as the dependent variable in trend analyses, with calendar year as the independent variable.

Changes over time were analyzed using one of seven statistical models, selected based on the nature of the data. The linear model (*y* = a + bty = a + bty = a + bt) assumes a constant rate of change over time. The quadratic model (*y* = a + bt + ct2y = a + bt + ct^2y = a + bt + ct2) and the cubic model (*y* = a + bt + ct2 + dt3y = a + bt + ct^2 + dt^3y = a + bt + ct2 + dt3) allow for non-linear trends, incorporating squared and cubic terms to capture more complex changes. In cases where an exponential relationship was suspected, the exponential model (ln(y) = a0 + bt\ln(y) = a_0 + btln(y) = a0 + bt) was used, transforming the dependent variable logarithmically. Similarly, the logarithmic model (*y* = a + b·ln(t)y = a + b\cdot \ln(t)y = a + b·ln(t)) was applied when changes in the predictor variable influenced the outcome in a decreasing manner, while the power model (ln(y) = a0 + b·ln(t)\ln(y) = a_0 + b \cdot\ln(t)ln(y) = a0 + b·ln(t)) was used when the relationship followed a power function. If no significant trend was detected, a constant model (y = ay = ay = a) was assumed, indicating stability over time. For each subgroup (sex and age), the best-fitting model was selected individually. For example, a linear model described the overall smoking prevalence trend, whereas a quadratic model best fit the 55-year-old male subgroup. The selection of the appropriate model was based on the coefficient of determination (*R*^2^), with consideration given only to models where the highest-order term was statistically significant. A significance level of 0.05 was applied, meaning that all *p*-values below this threshold were interpreted as indicating statistically significant relationships. The statistical analysis was performed using the R software.

## 3 Results

The overall smoking rate decreased from 28.91% in 2012 to 21.77% in 2021, representing a relative reduction of 24.6%, with the most substantial reduction observed in the 45-year age group (a relative decrease of 34.8%; 10.40 percentage points). Sex differences persisted throughout the study period, with males showing consistently higher smoking rates.

The prevalence of smoking varied significantly across age groups (*p* < 0.001). As shown in Table 1, older individuals consistently had a higher percentage of smokers compared to younger age groups. In 2012, smoking prevalence was highest among individuals aged 55 + years (34.47%) and lowest among those aged 35–39 years (24.60%). Over the study period, a general decline in smoking prevalence was observed across all age groups. By 2021, the proportion of smokers had decreased in each age category, with the most notable decline in the oldest group (from 34.47% in 2012 to 24.88% in 2021; a relative decrease of 27.8%). These findings suggest an overall reduction in smoking rates over time, with significant differences between age groups.

**Table 1:** Prevalence of smoking by age group (2012–2021).

Year	Smoking cigarettes	Group					*p*
		35 years (*N* = 21,087)	40 years (*N* = 19,057)	45 years (*N* = 15,458)	50 years (*N* = 15,027)	55 years (*N* = 13,345)	
2012	Yes	5,187 (24.60%)	4,906 (25.74%)	4,622 (29.90%)	4,786 (31.85%)	4,600 (34.47%)	*p* < 0.001
	No	15,900 (75.40%)	14,151 (74.26%)	10,836 (70.10%)	10,241 (68.15%)	8,745 (65.53%)	
2013	Yes	4,023 (23.71%)	3,446 (23.83%)	3,026 (27.03%)	3,320 (31.26%)	3,135 (32.54%)	*p* < 0.001
	No	12,948 (76.29%)	11,014 (76.17%)	8,169 (72.97%)	7,301 (68.74%)	6,498 (67.46%)	
2014	Yes	3,907 (22.97%)	3,758 (22.83%)	3,258 (25.67%)	3,331 (29.68%)	3,335 (32.41%)	*p* < 0.001
	No	13,105 (77.03%)	12,706 (77.17%)	9,433 (74.33%)	7,893 (70.32%)	6,954 (67.59%)	
2015	Yes	4,118 (22.84%)	4,062 (22.00%)	3,485 (24.40%)	3,506 (28.87%)	3,262 (30.68%)	*p* < 0.001
	No	13,908 (77.16%)	14,398 (78.00%)	10,798 (75.60%)	8,640 (71.13%)	7,371 (69.32%)	
2016	Yes	3,659 (21.94%)	3,911 (21.73%)	3,526 (24.75%)	3,150 (27.63%)	2,935 (30.46%)	*p* < 0.001
	No	13,016 (78.06%)	14,089 (78.27%)	10,723 (75.25%)	8,251 (72.37%)	6,702 (69.54%)	
2017	Yes	4,855 (22.12%)	4,666 (21.34%)	4,228 (23.31%)	3,697 (26.46%)	3,372 (28.76%)	*p* < 0.001
	No	17,096 (77.88%)	17,194 (78.66%)	13,910 (76.69%)	10,273 (73.54%)	8,351 (71.24%)	
2018	Yes	4,672 (22.60%)	4,180 (21.11%)	3,880 (22.79%)	3,296 (25.82%)	3,011 (28.73%)	*p* < 0.001
	No	16,005 (77.40%)	15,617 (78.89%)	13,146 (77.21%)	9,469 (74.18%)	7,470 (71.27%)	
2019	Yes	5,133 (22.58%)	4,962 (22.02%)	4,254 (21.78%)	3,679 (25.29%)	3,199 (28.34%)	*p* < 0.001
	No	17,598 (77.42%)	17,571 (77.98%)	15,278 (78.22%)	10,866 (74.71%)	8,087 (71.66%)	
2020	Yes	2,234 (21.36%)	2,305 (21.64%)	2019 (21.20%)	1719 (23.38%)	1,521 (27.26%)	*p* < 0.001
	No	8,223 (78.64%)	8,345 (78.36%)	7,505 (78.80%)	5,635 (76.62%)	4,058 (72.74%)	
2021	Yes	2,147 (21.05%)	2,270 (20.84%)	2019 (19.50%)	1754 (22.59%)	1,390 (24.88%)	*p* < 0.001
	No	8,052 (78.95%)	8,622 (79.16%)	8,335 (80.50%)	6,012 (77.41%)	4,196 (75.12%)	

*p* – the chi-square test.

Table 2 presents the prevalence of smoking among women in different age groups between 2012 and 2021. A statistically significant association was observed (*p* < 0.001), with older women generally exhibiting higher smoking rates. In 2012, the highest prevalence was noted among 50-year-old women (27.83%) and 55-year-old women (30.08%), while the lowest was in the 35-year-old group (19.97%).

**Table 2:** Prevalence of smoking among females by age group (2012–2021).

Year	Smoking cigarettes	Group					*P*
		35 years – Female (*N* = 13,239)	40 years – Female (*N* = 11,944)	45 years – Female (*N* = 9,572)	50 years – Female (*N* = 9,171)	55 years – Female (*N* = 7,980)	
2012	Yes	2,644 (19.97%)	2,464 (20.63%)	2,439 (25.48%)	2,552 (27.83%)	2,400 (30.08%)	*p* < 0.001
	No	10,595 (80.03%)	9,480 (79.37%)	7,133 (74.52%)	6,619 (72.17%)	5,580 (69.92%)	
2013	Yes	2081 (19.31%)	1773 (19.47%)	1,558 (22.26%)	1750 (26.71%)	1,616 (27.99%)	*p* < 0.001
	No	8,696 (80.69%)	7,331 (80.53%)	5,441 (77.74%)	4,801 (73.29%)	4,158 (72.01%)	
2014	Yes	1997 (18.64%)	1964 (18.74%)	1808 (21.93%)	1809 (25.73%)	1791 (28.56%)	*p* < 0.001
	No	8,715 (81.36%)	8,518 (81.26%)	6,435 (78.07%)	5,221 (74.27%)	4,481 (71.44%)	
2015	Yes	2,201 (19.17%)	2,188 (18.50%)	1916 (20.62%)	1898 (25.11%)	1735 (26.57%)	*p* < 0.001
	No	9,281 (80.83%)	9,640 (81.50%)	7,374 (79.38%)	5,660 (74.89%)	4,794 (73.43%)	
2016	Yes	1890 (17.89%)	2078 (18.11%)	1935 (20.96%)	1758 (24.22%)	1,571 (26.56%)	*p* < 0.001
	No	8,674 (82.11%)	9,396 (81.89%)	7,298 (79.04%)	5,499 (75.78%)	4,344 (73.44%)	
2017	Yes	2,493 (18.00%)	2,476 (17.87%)	2,295 (19.94%)	2000 (22.57%)	1775 (24.79%)	*p* < 0.001
	No	11,360 (82.00%)	11,382 (82.13%)	9,213 (80.06%)	6,860 (77.43%)	5,386 (75.21%)	
2018	Yes	2,424 (18.65%)	2,231 (17.78%)	2075 (19.06%)	1747 (21.68%)	1,563 (24.34%)	*p* < 0.001
	No	10,572 (81.35%)	10,318 (82.22%)	8,810 (80.94%)	6,311 (78.32%)	4,859 (75.66%)	
2019	Yes	2,701 (19.05%)	2,655 (18.79%)	2,319 (18.30%)	1996 (21.44%)	1,675 (23.95%)	*p* < 0.001
	No	11,475 (80.95%)	11,476 (81.21%)	10,351 (81.70%)	7,315 (78.56%)	5,319 (76.05%)	
2020	Yes	1,174 (17.56%)	1,206 (17.58%)	1,138 (18.37%)	919 (19.38%)	799 (23.23%)	*p* < 0.001
	No	5,513 (82.44%)	5,654 (82.42%)	5,058 (81.63%)	3,824 (80.62%)	2,641 (76.77%)	
2021	Yes	1,076 (16.84%)	1,209 (17.57%)	1,101 (16.53%)	970 (19.51%)	752 (21.44%)	*p* < 0.001
	No	5,315 (83.16%)	5,674 (82.43%)	5,558 (83.47%)	4,001 (80.49%)	2,755 (78.56%)	

*p* – the chi-square test.

Over the study period, smoking prevalence declined in all female age groups. By 2021, the proportion of smokers decreased to 10.76% in the youngest group (35 years; a relative reduction of 46.1%) and 21.44% in the oldest group (55 years; a relative reduction of 28.7%). These findings indicate a downward trend in smoking rates among women, with significant differences between age groups.

Table 3 presents the prevalence of smoking among male participants in different age groups between 2012 and 2021. A statistically significant association was observed (*p* < 0.001), with older males generally exhibiting higher smoking rates. In 2012, the highest prevalence was noted among 55-year-old males (41.01%) and 50-year-old males (38.15%), while the lowest was in the 35-year-old group (32.40%). Over the study period, smoking prevalence declined in all male age groups. By 2021, the proportion of smokers decreased to 28.12% in the youngest group (35 years; a relative reduction of 13.2%) and 30.69% in the oldest group (55 years; a relative reduction of 25.2%). These findings indicate a downward trend in smoking rates among males, with significant differences between age groups.

**Table 3:** Prevalence of smoking among male participants by age group.

Year	Smoking cigarettes	Group					P
		35 years – Male (*N* = 7,848)	40 years – Male (*N* = 7,113)	45 years – Male (*N* = 5,886)	50 years – Male (*N* = 5,856)	55 years – Male (*N* = 5,365)	
2012	Yes	2,543 (32.40%)	2,442 (34.33%)	2,183 (37.09%)	2,234 (38.15%)	2,200 (41.01%)	*p* < 0.001
	No	5,305 (67.60%)	4,671 (65.67%)	3,703 (62.91%)	3,622 (61.85%)	3,165 (58.99%)	
2013	Yes	1942 (31.35%)	1,673 (31.24%)	1,468 (34.99%)	1,570 (38.57%)	1,519 (39.36%)	*p* < 0.001
	No	4,252 (68.65%)	3,683 (68.76%)	2,728 (65.01%)	2,500 (61.43%)	2,340 (60.64%)	
2014	Yes	1910 (30.32%)	1794 (29.99%)	1,450 (32.60%)	1,522 (36.29%)	1,544 (38.44%)	*p* < 0.001
	No	4,390 (69.68%)	4,188 (70.01%)	2,998 (67.40%)	2,672 (63.71%)	2,473 (61.56%)	
2015	Yes	1917 (29.29%)	1874 (28.26%)	1,569 (31.42%)	1,608 (35.05%)	1,527 (37.21%)	*p* < 0.001
	No	4,627 (70.71%)	4,758 (71.74%)	3,424 (68.58%)	2,980 (64.95%)	2,577 (62.79%)	
2016	Yes	1769 (28.95%)	1833 (28.09%)	1,591 (31.72%)	1,392 (33.59%)	1,364 (36.65%)	*p* < 0.001
	No	4,342 (71.05%)	4,693 (71.91%)	3,425 (68.28%)	2,752 (66.41%)	2,358 (63.35%)	
2017	Yes	2,362 (29.17%)	2,190 (27.37%)	1933 (29.16%)	1,697 (33.21%)	1,597 (35.01%)	*p* < 0.001
	No	5,736 (70.83%)	5,812 (72.63%)	4,697 (70.84%)	3,413 (66.79%)	2,965 (64.99%)	
2018	Yes	2,248 (29.27%)	1949 (26.89%)	1805 (29.39%)	1,549 (32.91%)	1,448 (35.67%)	*p* < 0.001
	No	5,433 (70.73%)	5,299 (73.11%)	4,336 (70.61%)	3,158 (67.09%)	2,611 (64.33%)	
2019	Yes	2,432 (28.43%)	2,307 (27.46%)	1935 (28.20%)	1,683 (32.16%)	1,524 (35.51%)	*p* < 0.001
	No	6,123 (71.57%)	6,095 (72.54%)	4,927 (71.80%)	3,551 (67.84%)	2,768 (64.49%)	
2020	Yes	1,060 (28.12%)	1,099 (29.00%)	881 (26.47%)	800 (30.64%)	722 (33.75%)	*p* < 0.001
	No	2,710 (71.88%)	2,691 (71.00%)	2,447 (73.53%)	1811 (69.36%)	1,417 (66.25%)	
2021	Yes	1,071 (28.12%)	1,061 (26.47%)	918 (24.84%)	784 (28.05%)	638 (30.69%)	*p* < 0.001
	No	2,737 (71.88%)	2,948 (73.53%)	2,777 (75.16%)	2011 (71.95%)	1,441 (69.31%)	

*p* – the chi-square test.

## 4 Discussion

The CCRPP was developed specifically for adults at risk for CVD to enhance patients’ knowledge and awareness of cardiovascular disease and healthy behaviors, with the primary goal of achieving a 20% relative reduction in CVD incidence and mortality in the Polish population. We have shown that the CCRPP program has caused a significant reduction in the number of people smoking cigarettes in the years 2012–2021 (from 28.91% in 2012 to 21.77% in 2021). The largest reduction in cigarette smoking concerned men aged 45–55 (10–12%) and women aged 45 (almost 9%). Over the years 2012–2021, in each age group, the percentage of smokers was higher among men. Thus, smoking prevalence declined by an average of about 7% among CCRPP participants between 2012 and 2021, which may reflect broader public health trends or increasing health awareness in this group.

A notably sharp decline in smoking prevalence among 45-year-olds may reflect a confluence of factors beyond programmatic intervention. Mid-life is often a transitional stage accompanied by increased healthcare engagement for screening and chronic disease detection, which may have amplified cessation motivation. National registry data indicate that the prevalence of diagnosed hypertension is particularly high and rising in early middle-aged men, with the highest incidence observed in those aged 55–59, and elevated prevalence in younger men under 55 years (24). In parallel, trends in premature cardiovascular mortality in Poland during 2008–2017 show the greatest relative decreases among individuals aged 40–44, suggesting heightened public awareness and responsiveness to health messaging in neighboring age cohorts (25). These converging factors — enhanced detection of risk factors and greater salience of health risks at mid-life — likely synergized with CCRPP activity, contributing to the disproportionate decline in smoking at age 45.

The consistently higher smoking rates among men throughout the decade may be linked to multiple factors: cultural norms, occupational exposures, and differential coping strategies related to stress (26). Data from Poland show that smoking prevalence remains considerably higher in men than women, including a higher proportion of “hardcore” smokers—with 13.0% of men compared to 7.3% of women identified in a national survey (27). Tailored interventions—including workplace-based cessation programs, male-focused communication strategies, and integration of cessation support into routine occupational health assessments—may help address this disparity (26, 28).

Our study showed that conducting comprehensive CVD prevention programs led to a significant reduction in the incidence of one of the main cardiovascular (CV) risk factors. According to the World Health Organization (WHO), the percentage of smokers worldwide decreased from 22 to 17% between 2007 and 2021. Cigarette smoking contributes not only to an increased risk of CVD, but also to a number of other diseases including metabolic, oncological, respiratory, endocrine and gastrointestinal disorders (29–31). The 2021 European Society of Cardiology (ESC) guidelines on CVD prevention clearly indicate that smoking cessation should be recommended (class I recommendation) (32). In Poland, according to a study by Jankowski et al., in the group of women aged 40–49 and 50–59, the percentage of smokers is 33.3 and 27.6%, respectively. Among men the percentage is 35.6 and 37.5%, respectively (33). Thus, it appears that people covered by the CCRPP program are more motivated to quit smoking. It is estimated that every year, more than 82,800 adults in Poland are killed by tobacco-related diseases (34). Given that tobacco use is one of the most preventable causes of cardiovascular morbidity and mortality, reducing smoking rates remains a critical public health goal (34). Despite the countless data indicating the harmfulness of smoking, cigarettes have remained at the forefront of cardiovascular risk factors worldwide for decades. In 1990, smoking was in fifth place, while in 2019 it was in 6th place in terms of the prevalence of cardiovascular risk factors. Epidemiological data estimate indicate that in 2050 smoking will rank 7th in this ranking (35, 36). Although this trend is positive, the rate of decline remains too slow.

It is also important to acknowledge that the observed decline in smoking prevalence over the decade may not be solely attributable to the CCRPP program. Other contributing factors could include increased public awareness of the harms of smoking, broader health promotion initiatives, gradual shifts in social norms, and evolving patterns of nicotine product use. While our analysis suggests that the CCRPP played a meaningful role, particularly given the absence of new national tobacco regulations during this period, these additional influences should be considered when interpreting the findings. Furthermore, during the study period Poland continued to fulfill its obligations as a signatory to the WHO Framework Convention on Tobacco Control (FCTC) (37), which has been progressively strengthened over the past two decades. Although no major new national tobacco control strategy or legislation was developed, existing measures — including public smoking bans, graphic health warnings, and restrictions on tobacco advertising and sponsorship — remained in force, with periodic public health campaigns reinforcing these provisions (38). These ongoing efforts, aligned with FCTC guidelines, may have contributed to the observed decline in smoking prevalence, acting in parallel with the CCRPP and other health promotion activities (39–41). The observed decline in smoking prevalence, despite the absence of new national tobacco legislation, suggests that sustained, structured, primary care–based interventions such as the CCRPP can be an effective driver of behavioral change. By integrating cardiovascular risk assessment, personalized counseling, and follow-up into routine healthcare encounters, CCRPP may have provided continuous motivation and opportunities for cessation that complemented existing legislative measures.

Therefore, implementing comprehensive programs such as CCRPP on a large scale can contribute to a faster reduction in the number of smokers, not only in Poland but also worldwide. The results of the meta-analysis by Bakhit et al. (42) emphasize the key role of implementing comprehensive programs such as CCRPP in CVD prevention. This meta-analysis showed that providing patients with information about the benefits of quitting smoking, both in primary and secondary prevention, did not translate into a significant reduction in smoking (42). It is worth emphasizing that education should clearly indicate: (1) never starting smoking; (2) quitting smoking as soon as possible; (3) using new forms of smoking (e-cigarettes, heat-not-burn [HnB] systems) only as a way to quit smoking and not as a substitute. It has been shown that only smoking cessation, but not reduction, was associated with reduced CVD risk (12). Cessation of smoking significantly contributes to the reduction of CV risk compared to individuals who continue smoking. The extent of risk reduction is positively correlated with the duration of smoking abstinence, with longer periods of cessation leading to a progressive decline in CV risk. Over time, this risk approaches that of individuals who have never smoked; however, it does not fully revert to baseline levels, indicating a persistent residual risk attributable to prior tobacco exposure (43). Therefore, the best form of prevention is to never smoke cigarettes.

Despite the fact that our analysis did not include new forms of smoking: e-cigarettes and heated tobacco systems (HnB), it is worth paying attention to them, as some people may have switched from traditional cigarettes to these modern forms. Furthermore, these new forms of smoking, particularly disposable e-cigarettes, are currently the primary method of nicotine initiation among children and adolescents (44). Unfortunately, despite a partially better safety profile, e-cigarettes and HnB are often used at the same time (double and triple smokers) (45). Simultaneous use of 2–3 forms of smoking is associated with a significantly higher cardiovascular risk compared to smoking, for example, only traditional cigarettes (46). Taking this into account, educational programs should emphasize that, for example, e-cigarettes, in a similar way to traditional ones, increase the risk of CVD and stroke (46). The use of e-cigarettes and HnB systems is associated with an increase in cardiovascular risk (47, 48). Therefore, it is recommended to conduct a more extensive medical interview regarding cigarette smoking, as some people may not recognize e-cigarettes and HnB as cigarettes.

Conducting comprehensive smoking cessation programs, such as CCRPP, significantly increases the likelihood of successfully quitting tobacco use. To enhance cessation success rates, a structured approach should be implemented, incorporating motivation by highlighting the benefits of quitting, thorough assessment, and, if necessary, additional education and encouragement, with recognition of previous quit attempts made by the patient. An essential component of this process is the use of nicotine replacement therapy, along with support from close family members (49, 50). Evidence suggests that a gradual, step-by-step approach to smoking cessation, based on education and motivation, is more effective in achieving long-term abstinence compared to abrupt quitting attempts (51). It is also worth mentioning that different factors influence smoking cessation in women and men. The main barriers to smoking cessation in women were psychological factors, such as emotion and stress, compared with environmental factors in men (26). Women despite smoking fewer cigarettes and being less nicotine dependent than men, women find it more difficult to quit. Especially in the group of women, a multidisciplinary approach involving a psychologist, dietitian, and physical activity specialist is important in the process of quitting smoking (52).

This study offers added value beyond confirming global declines in smoking prevalence by providing a decade-long, large-scale, program-based analysis drawn from the CCRPP in Poland. Unlike previous national surveys, our dataset includes over 880,000 participants stratified into fixed age cohorts and analyzed separately by sex, allowing the identification of trends not previously described in the Polish context. These include the most pronounced decline in smoking among individuals aged 45 years, the persistence of significant sex disparities despite overall reductions, and the observation that such changes occurred in the absence of major new national tobacco control legislation. These findings demonstrate the potential for structured, long-term prevention programs embedded in primary healthcare to drive measurable behavioral change, offering a model that can be adapted and applied in other countries. From a policy perspective, our findings reinforce the need to scale structured prevention programs like CCRPP within primary care, especially targeting middle-aged men. National strategies could incorporate smoking cessation counseling into routine health checks, increase access to pharmacotherapy, and expand surveillance to include alternative nicotine products (53, 54). Public campaigns should also address gender-specific barriers to quitting, with tailored messaging that resonates with men’s motivations and contexts.

In summary, our research has shown that the comprehensive CVD prevention program – CCRPP has led to a significant reduction in the percentage of smoking women and men in Poland over the past decade. Nevertheless, the number of smokers is still too high and it is necessary to promote even more knowledge about the harmfulness of smoking (taking into account the increasingly available new forms of smoking, which are increasingly used by children).

### 4.1 Study limitations

This study has several limitations that should be considered when interpreting the results. First, the data were obtained from the CCRPP, which may introduce selection bias, as participants were likely more health-conscious and motivated to engage in preventive healthcare compared to the general population. Second, smoking status was self-reported, which may have led to reporting bias, as individuals might have underreported or misreported their smoking habits. Third, the study lacked detailed socioeconomic and clinical data, preventing an in-depth analysis of potential confounders such as income, education level, and family history of cardiovascular disease, which could have influenced smoking behavior trends. In particular, the CCRPP dataset did not capture key determinants such as socioeconomic status, urban/rural residency, or comorbidities, which limited our ability to adjust for these potential confounders or to examine disparities in smoking prevalence across different population subgroups. Moreover, the dataset was available only in aggregated form (annual prevalence by sex and age group), without individual-level records. This precluded the use of multivariable statistical approaches such as logistic regression with interaction terms (e.g., age × sex, time × age) and the calculation of regression-based confidence intervals. This study spans a decade (2012–2021), during which public health policies and smoking regulations were already well-established. While previously established smoking restrictions and tobacco control measures remained in effect, no significant new regulations were introduced during this period. However, healthcare access, public awareness campaigns, and the continued enforcement of national tobacco control measures under the WHO Framework Convention on Tobacco Control (FCTC) evolved during this period, potentially affecting smoking trends. As the CCRPP dataset did not include information on participants’ exposure to these broader interventions, we could not disentangle their effects from the program’s impact. The COVID-19 pandemic (2020–2021) likely impacted both participation rates and smoking behaviors among CCRPP participants. Lockdowns and disruptions in healthcare services limited program coverage, and pandemic-related stress may have increased smoking in some individuals. However, data in this area are inconsistent, with some suggesting an increase in smoking due to stress, while others suggest a decrease because smoking was perceived as a risk factor for COVID-19 (55, 56). It is worth noting that the use of new forms of smoking increased significantly during the COVID-19 pandemic (57). Conversely, heightened public concerns about respiratory health and COVID-19-related messaging may have prompted others to attempt to quit (58). These conflicting influences make it difficult to discern the true impact of the CCRPP on smoking prevalence in the most recent years of observation. Despite these limitations, the study provides valuable insights into temporal trends in smoking prevalence in Poland and underscores the effectiveness of structured prevention programs in reducing smoking rates. Future research should incorporate additional demographic and behavioral variables, as well as objective biomarkers, to enhance the understanding of smoking patterns and improve targeted intervention strategies. The present study focused exclusively on smoking status, despite the availability of other relevant variables in the CCRPP dataset, such as education, physical activity, and alcohol use. While this approach allowed for a detailed analysis of one key modifiable cardiovascular risk factor, it does not account for the potential influence of these other factors on smoking prevalence and trends. Future analyses could incorporate these variables to provide a more comprehensive picture of smoking behaviors in the Polish population.

## 5 Conclusion

In this nationwide, repeated cross-sectional study of the CCRPP, smoking prevalence declined markedly between 2012 and 2021, most notably in middle-aged adults. Persistent sex disparities highlight an urgent need for targeted cessation strategies for men. These findings support the continuation and expansion of structured prevention programs within primary care, with integration of demographic-specific interventions and systematic monitoring of emerging tobacco products. Public health policy should prioritize sustained investment in such initiatives, coupled with enhanced surveillance to capture socioeconomic and geographic disparities. Future research using individual-level, representative data is essential to determine the causal impact of these programs and to guide more equitable, effective tobacco control. From a policy perspective, our findings imply that embedding structured smoking cessation and cardiovascular risk reduction interventions within primary care can yield measurable public health benefits, even in the absence of new national legislation. Such models could be replicated in settings where legislative change is delayed or politically challenging. The lessons learned from CCRPP may inform and inspire similar large-scale, evidence-based initiatives in other countries facing persistent tobacco-related health burdens.

## Data Availability

The raw data supporting the conclusions of this article will be made available by the authors, without undue reservation.
